# Improving medication appropriateness in nursing homes via structured interprofessional medication-review supported by health information technology: a non-randomized controlled study

**DOI:** 10.1186/s12877-020-01895-z

**Published:** 2020-11-26

**Authors:** Johanna Katharina Dellinger, Stefan Pitzer, Dagmar Schaffler-Schaden, Maria Magdalena Schreier, Laura Sandre Fährmann, Georg Hempel, Rudolf Likar, Jürgen Osterbrink, Maria Flamm

**Affiliations:** 1 Institute of Nursing Science and Practice, Paracelsus Medical University, Salzburg, Austria; 2 Institute of General Practice, Family Medicine and Preventive Medicine, Paracelsus Medical University, Salzburg, Austria; 3 Institute of Pharmaceutic and Medical Chemistry, University of Münster, Münster, Germany; 4 Klinikum Klagenfurt am Wörthersee, Klagenfurt am Wörthersee, Austria

**Keywords:** Interprofessional medication review, Long term care, Interprofessional relations, Drug therapy, Potentially inappropriate medication

## Abstract

**Background:**

In nursing home residents (NHRs), polypharmacy is widespread, accompanied by elevated risks of medication related complications. Managing medication in NHRs is a priority, but prone to several challenges, including interprofessional cooperation. Against this background, we implemented and tested an interprofessional intervention aimed to improve medication appropriateness for NHRs.

**Methods:**

A non-randomized controlled study (SiMbA; “Sicherheit der Medikamentherapie bei AltenheimbewohnerInnen”, Safety of medication therapy in NHRs) was conducted in six nursing homes in Austria (2016–2018). Educational training, introduction of tailored health information technology (HIT) and a therapy check process were combined in an intervention aimed at healthcare professionals. Medication appropriateness was assessed using the Medication Appropriateness Index (MAI). Data was collected before (t0), during (t1, month 12) and after (t2, month 18) intervention via self-administered assessments and electronic health records.

**Results:**

We included 6 NHs, 17 GPs (52.94% female) and 240 NHRs (68.75% female; mean age 85.0). Data of 159 NHRs could be included in the analysis. Mean MAI-change was − 3.35 (IG) vs. − 1.45 (CG). In the subgroup of NHRs with mean MAI ≥23, MAI-change was − 10.31 (IG) vs. −3.52 (CG). The intervention was a significant predictor of improvement in MAI when controlled for in a multivariable regression model.

**Conclusions:**

Improvement of medication appropriateness was clearest in residents with inappropriate baseline MAI-scores. This improvement was independent of variances in certain covariates between the intervention and the control group. We conclude that our intervention is a feasible approach to improve NHRs’ medication appropriateness.

**Trial registration:**

DRKS Data Management, ID: DRKS00012246. Registered 16.05.2017 – Retrospectively registered.

## Background

Ensuring medication appropriateness in residents of long-term care facilities is known to be a challenge for all involved health care professionals [1, 2]. Age-related changes in pharmacokinetics and pharmacodynamics complicate the process of prescribing for this group of patients [3]. Additionally, residents of long-term aged care facilities often suffer from multiple co-morbidities [4], which can easily lead to complex medication regimes, even when guidelines for the appropriate treatment of individual diseases are followed and polypharmacy is appropriate [5].

In a systematic review on polypharmacy (defined as ≥5 concurrent medications) in nursing home residents (NHRs), prevalence in 11 international studies ranged from 38.1 to 91.2% [6]. In a 2016 study in Austrian nursing homes, prevalence of polypharmacy was between 74.1 and 79.1% [7]. In addition, polypharmacy is a well-documented risk factor for inappropriate medication [8–10].

Storms et al. conducted a systematic review and found the reported prevalence of inappropriate medication to vary between 18.5 and 79% in nursing homes residents, depending on the criteria used [11]. Based on the Austrian consensus list of potentially inappropriate medication (PIM) [12], the rate of NHRs in Austria prescribed at least 1 potentially inappropriate drug was 72,9% [7].

Inappropriate medication in NHRs increases the risk of adverse health outcomes, including deteriorations of the physical and cognitive status or preventable hospitalization and death [1, 4, 13]. Against this background, managing medication appropriateness in NHRs is a priority, but it is prone to several hindering factors concerning interprofessional coordination. Whilst interprofessional collaboration essentially has the potential to enhance efficiency, improve outcomes and respond to the growing complexity of caring for patients with complex morbidities [14], inadequate information exchange e.g. induced by ambiguous documentation systems, the lack of coordination mechanisms between health care providers and the use of communication channels with restricted communication bandwidth (like telephone and fax) can lead to gaps in the transfer of medication-related information and pose potential medication safety issues [15]. In an effort to bridge these gaps, health information technology (HIT) has been proposed and successfully used in several phases of medication management [16, 17]. However, since changes in communication technology are related to organizational change, success of HIT in enhancing interprofessional collaboration requires tailored integration into existing everyday care practice to prevent conflict over professional roles, impractical solutions through overemphasis on technological aspects and lack of personnel resources to acquire new skills in digitally mediated communication [14].

Given the challenges of polypharmacy and the relevance of interprofessional cooperation for medication appropriateness and the potential of HIT-solutions to support it, our study aimed to evaluate the effectiveness of an intervention, which targets the cooperation of professions involved in NHRs’ medication therapy and is supported by tailored HIT. We hypothesized that HIT-assisted specific education and structured multi-professional medication-review and -monitoring improves medication appropriateness in NHRs. The present paper examines this hypothesis.

## Methods

### Study type and setting

The SiMbA-study (“Sicherheit der Medikamentherapie bei AltenheimbewohnerInnen”, Safety of medication therapy in NHRs) was a non-randomized controlled study set in Austrian nursing homes between 2016 and 2018. It was supplemented with a qualitative evaluation of its intervention after the interventional period, which is not part of this paper and will be published elsewhere. The study protocol has been published previously [18] and the study was registered with the German Clinical Trials register (DRKS-ID: DRKS00012246).

Due to a priori known close structural and personal relationships within and between potentially participating nursing homes, we anticipated a risk of contamination bias if randomization were attempted on the individual level (NHR) or institutional level (NH). Therefore, each NH was assigned to either the control or intervention group based on an analysis of structural data (NH size, average care level, NHR-staff ratio, staff structure). This enabled us to match the two as good as possible. NHRs were blind to the group assignment, while participating healthcare professionals were not.

### Recruitment

Medication therapy in Austrian nursing homes involves three independent groups of health care professionals: General practitioners (GPs) primarily responsible for the prescription and monitoring of their patients’ medication, nurses who distribute medication and monitor intake and community pharmacies providing the prescribed medication. Consequently, recruitment included several steps: after selecting the NHs, recruitment started with GPs, as rejection rates were expected to be highest in this group. GPs were provided with information about the study and invited to participate. The patients of all GPs who had agreed to enroll in the SiMbA-study were then contacted and informed about the study. Inclusion criteria for NHR, given informed consent, were: age > 65, ≥1 prescriptions, not in quarantine (due to infections) or in an acute life-threatening situation.

Senior nursing home staff acted as gatekeepers and provided addresses for all GPs with patients in their facilities as well as pharmacies associated with the NHs. Participation of nursing staff and providing pharmacists depended on successfully recruiting at least one GP and his or her patients per nursing home.

### Intervention

The intervention aimed to improve medication appropriateness by enhancing and incorporating each professions particular expertise and capabilities within a standardized interprofessional medication review and monitoring process. It utilized HIT to create a formerly non-existent common information basis about NHRs for GPs, pharmacists and nurses, support standardization and foster accurate exchange of information. The intervention consisted of three steps:

1. Education: All healthcare professionals took part in a three-step-education addressing medication safety in older adults. Blending online and face-to-face training, it consisted of: a kick-off interprofessional face-to-face workshop (3 h), three profession-specific online sessions (each 20–45 min) with audio-visual presentations concluded by mandatory MC-tests and autonomous processing of case files addressing medication-related problems as well as a final interprofessional face-to-face event with instructions for the second part of the intervention (1.5 h). Pharmaceutical assistants as well as GPs’ receptionists were asked to attend the kick-off event, as we anticipated them to have great importance in the daily usage of the HIT-Tool SiM-Pl (SiMbA-Platform). Video footage of the event was provided to those participants who were not in attendance.
2. Integration of the HIT-Tool: SiM-Pl expanded the pre-existing electronic health record (EHR) for NHRs in the NH in three ways: First, SiM-Pl was designed to work on mobile devices, providing point-of-care-access. Second, it provides a secure log-in from outside of the internal network, allowing GPs and pharmacists to access the EHR from their respective workplaces for the first time (in the case of GPs) or at all (in the case of pharmacists). This means that for GPs, it functions as a computerized physician order entry (CPOE). Third, two add-ons were implemented: the TBB (“Therapie-Beobachtungsbogen”, Therapy monitoring form; assessment of adverse changes in health status), an assessment instrument used by nursing staff to monitor notable symptoms possibly related to present medication and medication change [19] and a medication review process [20]. All participants were provided with tablets (one tablet was provided per organization) and a token to generate secure logins for GPs and pharmacists. During the development of SiM-Pl, particular attention was paid to tailoring its features to the needs of the health care professionals intended to work with it.
3. Therapy check-process: A structured medication review and monitoring process was introduced (for further details, see the study protocol [18]). As a first step, participating GPs were asked to check the EHR with regard to drugs and diagnoses using their external login. Next, pharmacists performed a medication review for each NHR enrolled in the SiMbA project using the medication review tool, and at least one more before t1. Nurses were asked to complete a TBB for each participating NHR once every week or after a change in medication. Results of both were provided to the GPs in SiM-Pl upon log-in, in the form of reports. Communication between all three professional groups was possible with a direct messaging function. GPs were encouraged to make use of this to request individual medication checks at any time. The study team accompanied this process until t1, following up individually and reminding participants of their tasks in the process. Additionally, close technical support in form of a hotline was offered during this time.

### Measures and data collection

Medication appropriateness was measured using the Medication Appropriateness Index (MAI) [21]. The MAI covers ten aspects of medication appropriateness: indication, effectiveness, dosage, directions correct, directions practical, drug-disease interactions, drug-drug interactions, duplicates, duration and expense. For the SiMbA-study, item 10 (expense) was not included in the MAI, as the actual cost of individual medications could not be verified. This is due to the fact that pricing is arranged individually between nursing homes and pharmacies in Austria.

Against the background of literature, several variables to control for potential risk factors of inappropriate medication were assessed. These were age, sex, number of drugs prescribed, cognitive impairment (dementia screening scale (DSS) [22]), functional status (Katz Index of Independence in Activities of Daily Living (Katz ADL) [23, 24]) and comorbidities (Charlson Comorbidity Index (CCI) [25]).

Data was collected at baseline (t0), after 12 months at post-test (t1) and after 18 months at follow-up (t2) by (1) data export from the EHR (demographics and data for MAI application), (2) assessment by nurses familiar with NHR (Katz ADL, DSS) and (3) assessment by GPs (CCI).

The MAI was assessed by an independent clinical pharmacist (LF), using the medication data of participating NHRs as it was documented at the time of data collection. The pharmacist was blinded as to whether NHRs belonged to IG or CG. Based on the individual analysis of the prescribed medications, a weighted dichotomized score was determined for each NHR. Appropriate or marginally appropriate responses as well as “Don’t know” or “not applicable” were scored 0 for each item. Inappropriate responses were scored 1. This dichotomized score was then weighted in the following fashion: 3 for indication and effectiveness, 2 for dosage, directions, drug-drug interaction and drug-disease interaction and 1 for practical directions, duplication and duration [26]. For each NHR, the MAI-score was calculated combining the ratings of all items for all prescribed medications. Consequently, a higher MAI-score indicates less appropriate medication.

### Hypothesis and required sample size

Since there is neither an agreed upon value of MAI that signifies inadequate medication appropriateness nor a definition of what constitutes a meaningful improvement of medication appropriateness (e.g. [27]), a combined approach was used to define minimal important change of MAI. First, we looked at the MAI itself. Items most relevant to medication appropriateness are weighted the highest (“3”). Accordingly, a change of 3.00 MAI-points can be interpreted as a meaningful improvement, as it corresponds to change in one highly weighted dimension of the MAI. Second, we adopted a statistical point of view by defining the minimal relevant change as a difference amounting to at least half the estimated SD of the outcome [28]. Using Crotty et al. [29] as reference population, this value was calculated to be 3.00. Consequently, a reduction of 3.00 MAI points was defined as the minimally relevant change for the purposes of this study.

We hypothesized that the mean change in the value of the MAI of NHRs between t1 and t0 in the intervention group differs by at least 3.00 MAI points from the mean change in the control group. Additionally, we expected this improvement to persist at the third data collection point (t2).

Required sample size was calculated for the minimal important difference of MAI as *n* = 29 per group (α = 0.05; β = 0.20). Considering an expected drop-out rate of 37% (death of residents during study period, refusal to stay in the study), to reach *n* = 29 at t2 required a sample size of 47 per group at t0. Since refusal to participate in the study was conservatively assumed to be 75% in contacted physicians and 50% in contacted residents, we planned to contact GPs caring for a total of 369 NHR, to ultimately reach *n* = 47 at t0.

### Analysis

Analysis of the MAI focused on changes of MAI scores for *long-term medication* between t0 and [26, 30]. A variable for the mean change within-group for t1-t0 was calculated and analyzed descriptively. Additionally, effect sizes were calculated for within-group differences (d_cohen_) [31] as well as for between-group differences (d_ppc2_) [32].

Recent studies have found a greater potential for change in medication appropriateness when baseline appropriateness is comparatively poor [27], with one study not finding enough scope for improvement when MAI scores were low to begin with [33]. However, there is no established cut-off point for a MAI value indicating “poor” medication appropriateness. One study found a cut-off value of 24 at baseline to define the subgroup expected to show a substantially larger benefit from an intervention targeting medication appropriateness [27]. We identified a cut-off value for the intervention group in our sample using the same technique, namely a ROC-curve based on the minimally important difference of − 3.00. A descriptive comparison of changes in the subgroups above and below cut-off was performed.

The robustness of the results from the descriptive MAI-analysis was tested via multivariable linear regression. Mean MAI-change between t0 und t1 was the outcome variable. A dichotomized variable representing the intervention (intervention group = 1) was used as the main predictor; additionally, age, sex (dichotomized, female = 1), CCI (continuous), baseline MAI score (continuous), functional status (Katz ADL, continuous) and cognitive status (DSS, continuous) were included in the regression model. Number of drugs was exchanged for the baseline MAI (as a continuous variable), since the appropriateness of medication at baseline differed significantly between groups and we also expected it to be an important predictor of change (the higher the baseline MAI, the higher the potential change). Model diagnostics were performed.

Statistical analysis was performed using IBM®SPSS Statistics 24.0.

## Results

**Fig. 1:**
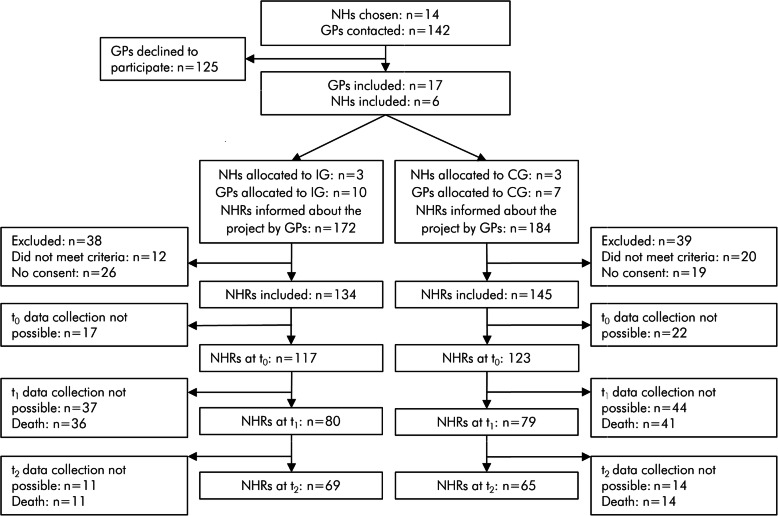
Flow chart of recruitment process and data collection. Reasons for drop-out: death, hospitalization, relocation, withdrawal of consent

Six of twelve NHs participated in the study. Within those 6 NHs, 17 of 142 contacted GPs, and 3 of 14 contacted pharmacies participated in the study (see Fig. 1 for details on the recruitment process). Twenty-four of twenty-eight healthcare professionals participating in the study were present at the interprofessional kick-off-event. Individualized access keys to the profession-specific online education sessions were provided to all healthcare professionals, 26 of 28 completed the educational intervention. One GP not able to attend the final face-to-face event was instructed personally with regards to the therapy check process. Overall, 233 medication checks were performed by pharmacists over the course of the study, and 2698 TBBs were completed by nurses. No substantial differences between NHs of the IG in applying these measures were observed.

Data on 240 NHRs was collected at baseline (t_0_) (depicted in Table 1). At baseline, the intervention group was on average significantly younger, had a significantly higher co-morbidity burden and a significantly lower baseline MAI than the control group. No significant mean differences were found between NHRs in sex, cognitive status and numbers of regularly prescribed drugs.

**Table 1:** Characteristics of Nursing Home Residents (NHR) at baseline (t_0_)

	Total sample (*n =* 240*)*		Subgroup MAI_**t0**_ < 23 (*n =* 109)		Subgroup MAI_**t0**_ ≥ 23 (*n =* 131)	
	IG	CG	IG	CG	IG	CG
	(*n* = 117)	(*n =* 123)	(*n* = 65)	(*n* = 44)	(*n* = 52)	(*n =* 79)
**Age**						
M ± SD	83.44^a^ ± 8.13	86.42^a^ ± 7.96	85.1^b^ ± 7.0	86.5 ± 8.0	81.4^a^ ± 9.0	86.38^a^ ± 7.99
Min - Max	65–97	66–102	67–97	68–96	65–97	66–102
**Sex**						
Female n (%)	74 (63.25)	91 (73.98)	42 (64.62)	36 (81.82)	32 (61.54)	55 (69.62)
**Cognitive Status**						
DSS-score (0–14), M ± SD	5.56 ± 4.52	4.47 ± 4.75	6.57 ^b^ ± 4.54	6.04 ± 4.78	4.33 ^b^ ± 4.21	3.78 ± 4.60
	(*n =* 109)	(*n =* 91)	(*n = 60*)	(*n = 28*)	(*n =* 49)	(*n =* 63)
**Comorbidities**						
CCI M ± SD	4.6^a^ ± 2.4	3.3^a^ ± 2.1	4.66 ± 2.24	2.47 ± 1.52	4.57 ± 2.64	3.78 ± 2.23
Min – Max	0–10	0–10	0–10	0–6	0–10	0–10
Dementia n (%)	89 (82.41)	85 (75.89)	54 (87.10)	27 (72.97)	35 (76.09)	58 (77.33)
Cerebrovascular Disease n (%)	81 (75.00)	74 (66.07)	49 (79.03)	27 (72.97)	32 (69.57)	47 (62.67)
Congestive Heart Failure n (%)	53 (49.07)	31 (27.68)	29 (46.77)	6 (16.22)	24 (52.17)	25 (33.33)
	(*n* = 106)	(*n* = 112)	(*n = 59*)	(*n = 38*)	(*n* = 47)	(*n* = 74)
**Functional Status**						
Katz ADL (0–6) M ± SD	2.1 ± 2.1	2.3 ± 2.1	1.86 ± 2.08	1.98 ± 2.03	2.30 ± 2.21	2.47 ± 2.18
	(*n =* 114)	(*n =* 122)	(*n = 64*)	(*n =* 43)	(*n =* 50)	(*n = 79*)
**Number of regularly prescribed drugs**						
Per NHR M ± SD	9.6 ± 4.4	10.7 ± 4.7	7.43 ^b^ ± 3.56	7.36 ± 3.40	12.33 ± 3.78	12.58 ± 4.29
Min – Max	2–23	1–26	2–16	1–18	7–23	6–26
≥ 5 drugs n (%)	101(86.32)	114 (92.68)	49 (75.38)	35 (79.55)	52 (100)	79 (100)
≥ 10 drugs n (%)	55 (47.01)	73 (59.35)	16 (24.62)	10 (22.73)	39 (75.00)	63 (79.75)
**Baseline MAI**						
Of regular drugs M ± SD	24.55^a^ ± 16.19	30.8^a^ ± 16.3	12.82^a b^ ± 5.80	15.25^a^ ± 4.83	39.21 ± 12.63	39.53 ± 13.80
Min – Max	2–73	2–80	2–22	2–22	23–73	23–80

Notes: *IG* intervention group, *CG* control group, *M* Mean, *SD* Standard deviation, *wMAI* Weighted MAI Sum score for long-term medication; Subgroup wMAIt_0_ < 23 = subgroup of NHRs with a wMAI score < 23 at baseline; Subgroup wMAIt_0_ ≥ 23 = subgroup of NHRs with a wMAI score ≥ 23 at baseline

*DSS* Dementia Screening Score, *CCI* Charlson Comorbidity Index, *Katz ADL* Katz Index of Independence in Activities of Daily Living, *MAI* Medication Appropriateness Index

^a^ difference between IG and CG significant in t-test for independent samples

^b^ difference between subgroups in IG significant in t-test for independent samples

Calculation of the cut-off for comparatively poor medication appropriateness resulted in a MAI value of 23 (69.8 Sensitivity & 76.2 Specificity). In the subgroup with a baseline MAI above or equal to cut-off (MAI_t0_ ≥ 23), mean age was the only significant difference between the intervention and the control group, with the intervention group being significantly younger.

Comparing the two subgroups in the intervention group, NHRs in the subgroup below cut-off were significantly older, less cognitively able, had fewer medications regularly prescribed and a lower baseline MAI.

### Outcome

Medication appropriateness at baseline was worse in the control group than in the intervention group (see Table 2). Mean change in the intervention group is 1.9 MAI points larger than in the control group. Cohen’s d for between group-differences showed no effect (d_ppc2_ = − 0.09). Mean change in MAI scores between t0 and t1 was − 3.35 in the intervention group and − 1.45 in the control group. This equals a small effect in the intervention group, and no effect in the control group.

**Table 2:** Development of Medication Appropriateness Index (MAI) scores over the course of the SiMbA-study (t0-t2)

			t_0_	t_1_	t_2_	Mean difference t_1_-t_0_ (CI_95%_)	Effect size d_cohen_	Effect size d_ppc2_
IG	Total sample	M ± SD	24.55 ± 16.19 (*n =* 117)	21.16 ± 14.76 (*n =* 80)	21.39 ± 13.90 (*n =* 69)	− 3.35 (− 6.13; − 0.57)	d = − 0.22	Total sample: d_ppc2_ = − 0.09
	Subgroup wMAIt_0_ < 23	M ± SD	12.82 ± 5.80 (*n* = 65)	14.51 ± 9.94 (*n* = 45)	15.38 ± 9.36 (*n =* 37)	2.07 (− 0.60; 4.74)	d = 0.21	Subgroup wMAIt0 < 23: d_ppc2_ = − 0.14
	Subgroup wMAIt_0_ ≥ 23	M ± SD	39.21 ± 12.63 (*n =* 52)	29.71 ± 15.64 (*n =* 35)	28.34 ± 15.13 (*n =* 32)	− 10.31 (− 14.82; − 5.81)	d = − 0.67	
CG	Total sample^a^	M ± SD	30.87 ± 16.39 (*n =* 122)	28.97 ± 13.93 (*n =* 78)	28.58 ± 14.65 (*n =* 64)	−1.45 (− 3.79; − 0.89)	d = − 0.12	
	Subgroup wMAIt_0_ < 23	M ± SD	15.25 ± 4.83 (*n* = 44)	17.68 ± 8.45 (*n =* 28)	18.21 ± 11.27 (*n* = 24)	2.25 (− 0.13; − 4.63)	d = 0.36	Subgroup wMAIt_0_ ≥ 23: d_ppc2_ = − 0.38
	Subgroup^a^ wMAIt_0_ ≥ 23	M ± SD	39.68 ± 13.83 (*n =* 78)	35.30 ± 12.33 (*n =* 50)	34.80 ± 12.88 (*n =* 40)	− 3.52 (− 6.84; − 0.20)	d = − 0.34	

Notes: *IG* Intervention group, *CG* control group, *M* Mean, *SD* Standard deviation, *wMAI* Weighted MAI Sum score for long-term medication; Subgroup wMAIt_0_ < 23 = subgroup of NHRs with a wMAI score < 23 at baseline; Subgroup wMAIt_0_ ≥ 23 = subgroup of NHRs with a wMAI score ≥ 23 at baseline; d_cohen_ = Cohen’s d [31]; d_ppc2_ = effect size for pretest-posttest-control group design using pooled pretest SD [32]

^a^One extreme outlier (mean difference t_1_-t_0_ = 53) was excluded to match the sample in the regression (see Table 3)

Analysis of the subgroup above/equal to the cut-off showed that mean change in the intervention group was 6.79 MAI points larger than in the control group. Cohen’s d for between-group-differences gives this effect as d_ppc2_ = − 0.38. We observed a mean MAI reduction of − 10.31 in the intervention group, which fell further at t2, and a mean reduction of − 3.52 in the control group, with a further slight drop at t2. Effect sizes for these changes show a markedly larger effect in the intervention group than in the control group.

In the subgroup below cut-off, a slight increase of around 2.00 MAI points was observed in both intervention and control group.

### Regression

The intervention was no relevant predictor in the simple linear model (see Table 3, Model 1 and 2); introducing the various controls resulted in an increase of the correlation observed, showing a very small correlation and an estimated mean reduction of 3.53 MAI points for members of the intervention group. Only baseline MAI and the intervention correlated at all, with an estimated mean reduction of MAI by 0.35 MAI points per one-point increase in baseline MAI. Adjusted R^2^ was 0.20, so the model explained 20.4% of the variance in MAI change.

**Table 3:** Linear Regression-Models of MAI Change t_1_- t_0_ in Total Sample and Subgroup wMAIt0 ≥ 23

MAI change t_1_- t_0_	Model 1		Model 2	
Total sample *n* = 136	b (SE)	β (p)	b (SE)	β (p)
Constant	−1.87 (1.46)		13.23 (10.2)	
Intervention	−1.18 (1.99)	−0.05 (0.55)	−3.53 (2.0)	− 0.15 (0.08)
Baseline MAI	–	–	−0.35 (0.06)	**− 0.50 (0.00)**
Age	–	–	−0.07 (0.12)	− 0.05 (0.53)
Female	–	–	0.26 (2.0)	0.01 (0.90)
CCI	–	–	−0.03 (0.42)	0.01 (0.94)
Katz ADL	–	–	0.42 (0.46)	0.08 (0.36)
DSS	–	–	0.12 (0.23)	0.05 (0.59)
R^2^	0.00		0.20^a^	
	Model 3		Model 4	
Subgroup wMAIt0 ≥ 23 *n* = 73	b (SE)	β (p)	b (SE)	β (p)
Constant	−4.07 (1.87)		9.06 (16.25)	
Intervention	−5.99 (2.82)	**−0.24 (0.04)**	−6.31 (3.02)	**−0.26 (0.04)**
Baseline MAI	–	–	−0.36 (0.13)	**−0.38 (0.01)**
Age	–	–	−0.13 (0.18)	−0.09 (0.50)
Female	–	–	3.85 (3.20)	0.15 (0.23)
CCI	–	–	0.33 (0.66)	0.07 (0.61)
Katz ADL	–	–	0.94 (0.69)	0.18 (0.17)
DSS	–	–	0.36 (0.36)	0.13 (0.32)
R^2^	0.06		0.12^a^	

Notes: b = unstandardized regression coefficient; SE = standard error; β = standardized regression coefficient. wMAI = Weighted MAI Sum score for long-term medication; CCI = Charlson Comorbidity Index; KATZ ADL = Katz Index of Independence in Activities of Daily Living; DSS = Dementia Screening Score; Subgroup wMAIt0 ≥ 23 = subgroup of NHRs with a wMAI score ≥ 23 at baseline; Bolded βs are statistically significant (*p* < 0.05)

^a^ adjusted R^2^

Model diagnostics (outliers and influential cases, multicollinearity, homoscedasticity, normal distribution of errors) were performed. 1 extreme outlier (part of CG, change in MAI t_1_- t_0_ = 53) was excluded. Standardized residuals for this case were 4.39 (model 1), 4.62 (model 2), 4.06 (model 3) and 3.75 (model 4). Including the case risks overestimation of the intervention effect (if included, model 1 shows a correlation (β - 0.11 (0.19)) and β in model 2 is statistically significant; model 3 and 4 show only marginal increases in both strength of correlation and significance). Additionally, inclusion leads to non-normal distribution of errors in model 2 and slightly worse overall fit of the model

In the subgroup MAI_t0_ ≥ 23 (see Table 3, Model 3 and 4), the intervention proved to be both a relevant and a significant predictor (b = −.24, *p* < .05) in the simple linear model, with an estimated mean reduction of 6 MAI points in the intervention group. The multivariable model resulted in a similar estimate and a small significant correlation. Again, baseline MAI was the strongest predictor, a reduction of on average 0.36 MAI points estimated per one-point increase in baseline MAI. In the subgroup, the intervention remained both a relevant and significant predictor of mean MAI-change when controlled for the covariates baseline MAI, age, sex, comorbidities, independence of daily living and cognitive status.

The multivariable linear regression model for the subgroup MAIt0 < 23 (see Table 4) showed issues with model diagnostics; no relevant predictors for MAI change were found, resulting in a model with no predictive power.

**Table 4:** Linear Regression Models of MAI Change t_1_- t_0_ in Subgroup wMAIt0 < 23

MAI change t_1_- t_0_	Model 5		Model 6	
Subgroup wMAIt0 < 23 *n* = 63	b (SE)	Β (p)	b (SE)	Β (p)
Constant	2.23 (1.81)		19.62 (12.99)	
Intervention	0.19 (2.24)	0.01 (0.93)	0.69 (2.77)	0.04 (0.81)
Baseline MAI	–	–	−0.11 (0.20)	− 0.08 (0.57)
Age	–	–	−0.13 (0.15)	− 0.12 (0.38)
Female	–	–	−0.25 (2.59)	− 0.12 (0.39)
CCI	–	–	−0.62 (0.54)	− 0.17 (0.26)
Katz ADL	–	–	−0.19 (0.58)	−0.05 (0.75)
DSS	–	–	−0.11 (0.27)	−0.06 (0.68)
R^2^	0.00		−0.05^a^	

Notes: b = unstandardized regression coefficient; SE = standard error; β = standardized regression coefficient; p = significance; Bolded βs are statistically significant (*p* < 0.05); MAI = Weighted MAI Sum score for long-term medication; CCI = Charlson Comorbidity Index; KATZ ADL = Katz Index of Independence in Activities of Daily Living; DSS = Dementia Screening Score; Subgroup wMAIt0 < 23 = subgroup of NHRs with a wMAI score < 23 at baseline

^a^ adjusted R^2^

Model diagnostics show issues with model assumptions; there is heteroscedasticity for the predictor “intervention”, and distribution of errors is not normal based on the K-S-test. The model doesn’t fit the data well, R^2^ shows the model doesn’t explain variability in MAI change

## Discussion

The SiM-Pl-intervention with its combination of education, HIT and a structured medication check process, was successful in achieving a MAI reduction in the intervention group. The reduction in the intervention group exceeded the 3.00 MAI-points (− 3.3 [− 6.1; − 0.6]) considered a meaningful change, which was not achieved in the control group. Due to the MAI reduction in the control group, the difference in mean change between IG and CG was − 1.9 MAI points. This is less than the mean difference of − 3.88 observed in 5 RCTs using the MAI as outcome measure [34] and < 3.00 as stated in our hypothesis.

While our hypothesis was not confirmed for the whole sample, subgroup analysis showed substantial improvement of medication appropriateness in the intervention group: In line with expectations, change was generally more pronounced in the subgroup above the cut-off point of ≥23 MAI points at baseline. It just exceeded the 3 MAI-point-threshold of minimally important difference in the control group, but was nearly three times this difference at a reduction of 10.3 points in the intervention group. The mean difference of change is 6.8, which equals to a substantial improvement.

In terms of baseline characteristics, the intervention and the control group differed significantly from one another in that the intervention group was younger on average (mean age 83.44 ± 8.13 vs. 86.42 ± 7.96) and had a higher comorbidity burden (CCI mean 4.6 ± 2.4 vs. 3.3 ± 2.1) than the control group. Based on a systematic review of the literature, younger age is indicative of a higher risk for the use of inappropriate medication in NHRs [35], while high comorbidity was inconclusively significant in NHRs and found to be a risk factor in hospitalized patients. Following these baseline characteristics, we would cautiously expect medication appropriateness at baseline to be worse in the intervention group. The opposite is true, with baseline MAI significantly lower in the intervention group than in the control group (MAI mean 24.5 ± 16.2 vs. 30.8 ± 16.3). As a higher baseline MAI is associated with more potential for improvement [27], it is likely that the possible impact of the intervention is underestimated when based on a descriptive analysis of our sample. The multivariable regression model 2 (shown in Table 3), performed to address this issue, shows that predictive power of the intervention increases when the other factors are controlled for. This suggests baseline difference between the two groups may have obscured the effect of the intervention in the whole sample. Using the cut-off value of ≥23 MAI points leads to a better overall match between IG and CG (see Table 1).

A relevant issue with regards to monitoring medication appropriateness is being able to discern who might benefit from an intervention like this. As Hanlon & Schmader [30] pointed out, performing the MAI is very time consuming, which makes a cut-off point impractical for clinical practice, so several studies have tried to identify risk factors. We found that in the intervention group, NHRs in the group with ≥23 MAI points were significantly younger, more cognitively able and had more medications regularly prescribed. These findings are in line with results from Nothelle et al. [35], who describe younger age, comparably higher cognitive ability and number of medication as factors associated with inappropriate medication use in NHRs.

### Limitations

Our study has several limitations. First, it proved difficult to recruit GPs. This might have resulted in a bias towards medication regimes already closely monitored; motivated GPs who agreed to participate may already have had a larger interest in the topic medication safety. We could hypothesize that the effect of the intervention might have been larger if all physicians were included as less motivated physicians may reveal larger deficits in patient care thus giving a larger potential for improvement. Alternatively, the intervention could have a lower effect in a less motivated group of physicians. Second, given the fact that our analysis is based on routine data, it is difficult to differentiate if a diagnosis is missing due to documentation errors or if the indication is really not given. However, this mainly concerns the comparability of absolute numbers in MAI from our study with other studies. In our results, we focus on the mean change in MAI over the course of the study, and this should be sound given the fact that this limitation was present over the course of the entire study. Nevertheless, missing documentation of indication equals missing indication information for all health care professionals involved (except the prescriber) and is a medication safety issue in itself [15]. Third, the MAI is a surrogate endpoint, with no direct clinical implications. However, considering the high rate of comorbidities and mortality rates in the study population, defining clinical endpoints is difficult and may produce unreliable results. The MAI has the advantage of being a validated parameter.

## Conclusion

Since eMedication as well as increasing reliance on e-solutions in the health care-system are expected to gain importance in the next years, the implementation of HIT will be increasingly common. Whilst this goes along with various opportunities to support and enhance the benefit of structured interprofessional cooperation for appropriate medication in NH, implementation of HIT is a highly complex process, which has to be tailored to the professional and cultural characteristics of the specific health care teams involved to prevent major pitfalls [14].

In our study sample, we could demonstrate that the combined intervention in the SiMbA-study including education and an institutionally tailored HIT vehicle (SIM-Pl) for equally providing useful information to health professionals and establishing standards for interprofessional medication-review and -monitoring has a small effect regarding improvement of the MAI. However, the effect is explicitly larger within the subgroup of NHRs above the cut-off of ≥23 MAI points at baseline. It remains subject to further studies to define applicable criteria for daily routine to identify the subgroup that could benefit the most from intensified structured medication reviews. Though we could not identify the specific contribution of each single interventional measure, data let us conclude that such an intervention is a feasible approach to improve nursing home residents’ medication appropriateness.

## Data Availability

The datasets used and analysed during the current study are available from the corresponding author on reasonable request.

## Abbreviations

b: Unstandardized regression coefficient
β: Standardized regression coefficient
CCI: Charlson Comorbidity Index
CG: Control group
CI: Confidence Interval
CPOE: Computerized physician order entry
DSS: Dementia screening scale
EHR: Electronic health record
GP(s): General practitioner(s)
HIT: Health information technology
IG: Intervention group
Katz ADL: Katz Index of Independence in Activities of Daily Living
M: Mean
MAI: Medication Appropriateness Index
MC: Multiple choice
NH(s): Nursing home(s)
NHR(s): Nursing home resident(s)
PIM: Potentially inappropriate medication
SD: Standard deviation
SE: Standard error
SiMbA: “Sicherheit der Medikamentherapie bei AltenheimbewohnerInnen”, Safety of medication therapy in NHRs
SiM-Pl: SiMbA-Platform
TBB: “Therapie-Beobachtungsbogen”, Therapy monitoring form
wMAI: Weighted MAI Sum score for long-term medication
wMAIt_0_ < 23: Subgroup of NHRs with a wMAI score < 23 at baseline
wMAIt_0_ ≥ 23: Subgroup of NHRs with a wMAI score ≥ 23 at baseline
